# Updated review on novel therapies and ongoing clinical trials for high-risk non-muscle invasive bladder cancer

**DOI:** 10.3389/fonc.2025.1519428

**Published:** 2025-02-21

**Authors:** Brett Wiesen, Paige Hargis, Hunter Flores, Janet Kukreja

**Affiliations:** Division of Urology, Department of Surgery, University of Colorado Anschutz Medical Center, Aurora, CO, United States

**Keywords:** non muscle invasive bladder cancer, novel therapies, clinical trials, review, BCG (Bacille Calmette-Guérin)

## Abstract

**Purpose of review:**

The treatment options for high-risk non-muscle invasive bladder cancer (NMIBC), particularly in the setting of BCG-unresponsive disease, remain limited. We provide updates on recent, promising trials for high-risk NMIBC and newly FDA approved therapies.

**Recent findings:**

Several therapies with diverse mechanisms of action have shown favorable results in both BCG-naïve and BCG-unresponsive settings for NMIBC. These treatments include intravenous and intravesical immunotherapies, viral- and bacterial-based intravesical therapies, combination intravesical chemotherapy regimens, and novel methods of intravesical chemotherapy administration. Overall, the efficacy and tolerability of these emerging treatments for NMIBC appear promising, offering potential alternatives to radical cystectomy. There have also been recent FDA approvals for novel combination therapy for NMIBC which have been detailed below.

**Summary:**

As the landscape of managing BCG-unresponsive disease evolves, clinical trials will continue to expand the treatment options available for NMIBC.

## Introduction

Bladder cancer is the tenth most common cancer worldwide, with an incidence rate of almost half a million per year (1). Pathological stage and grade are the most important determinants of prognosis and treatment for bladder cancer (2, 3). Non-muscle invasive bladder cancer (NMIBC) is defined as cancer confined to the bladder mucosa and submucosa. NMIBC constitutes 75% of bladder cancer cases. Non-muscle invasive bladder cancer includes papillary tumors within the mucosa (stage Ta), tumors invading the lamina propria (stage T1), and flat high-grade lesions referred to as carcinoma *in situ* (CIS) (4). The term “non–muscle-invasive bladder cancer” presents an overall group definition, and all tumors must be defined according to their T-stage and histological grade. The cancer is considered muscle invasive if it is present in the muscularis propria of the specimen. There are multiple important distinctions regarding bladder cancer but the level of invasion of the tumor largely dictates the disease management. As opposed to muscle invasive cancer, NMIBC is primarily managed with local endoscopic/intravesical therapy and surveillance.

Transurethral resection of the bladder tumor (TURBT) in combination with intravesical chemotherapy or immunotherapy is considered standard therapy for NMIBC. However, despite intravesical treatment, patients are still at risk for tumor recurrence and progression to MIBC (5, 6). As a result of the great heterogeneity seen in NMIBC, a risk stratification system has been adopted to classify NMIBC to either low-, intermediate-, or high-risk. The tumors are stratified into risk groups that account for known risk factors of cancer progression and recurrence, including tumor stage, grade, size, focality, presence of CIS, lymphovascular invasion, recurrence rate, and response to intravesical treatments (7–9). Definitions of high-risk NMIBC according to the American and European Urologic Associations are detailed in **Table 1**.

**Table 1:** Definitions of high-risk non-muscle-invasive bladder cancer from the American Urologic Association and the European Urologic Association.

American Urologic Association	European Urologic Association
Carcinoma *in situ*	**HIGH RISK** Carcinoma *in situ*
High-grade T1 tumors	Any high-grade tumor
Recurrent or multifocal or large (> 3 cm) high-grade Ta tumors	Any T1 tumor
Any tumor following BCG failure	Multiple, recurrent, and large low-grade Ta tumors
Lymphovascular invasion	**HIGHEST RISK** T1 High grade with CIS
Non-Urothelial Histology	Multiple, large, or recurrent T1 high-grade tumors
High-grade tumor involving the prostatic urethra	T1 with CIS in prostatic urethra
	Some variant histology
	Lymphovascular invasion

**Figure 1:**
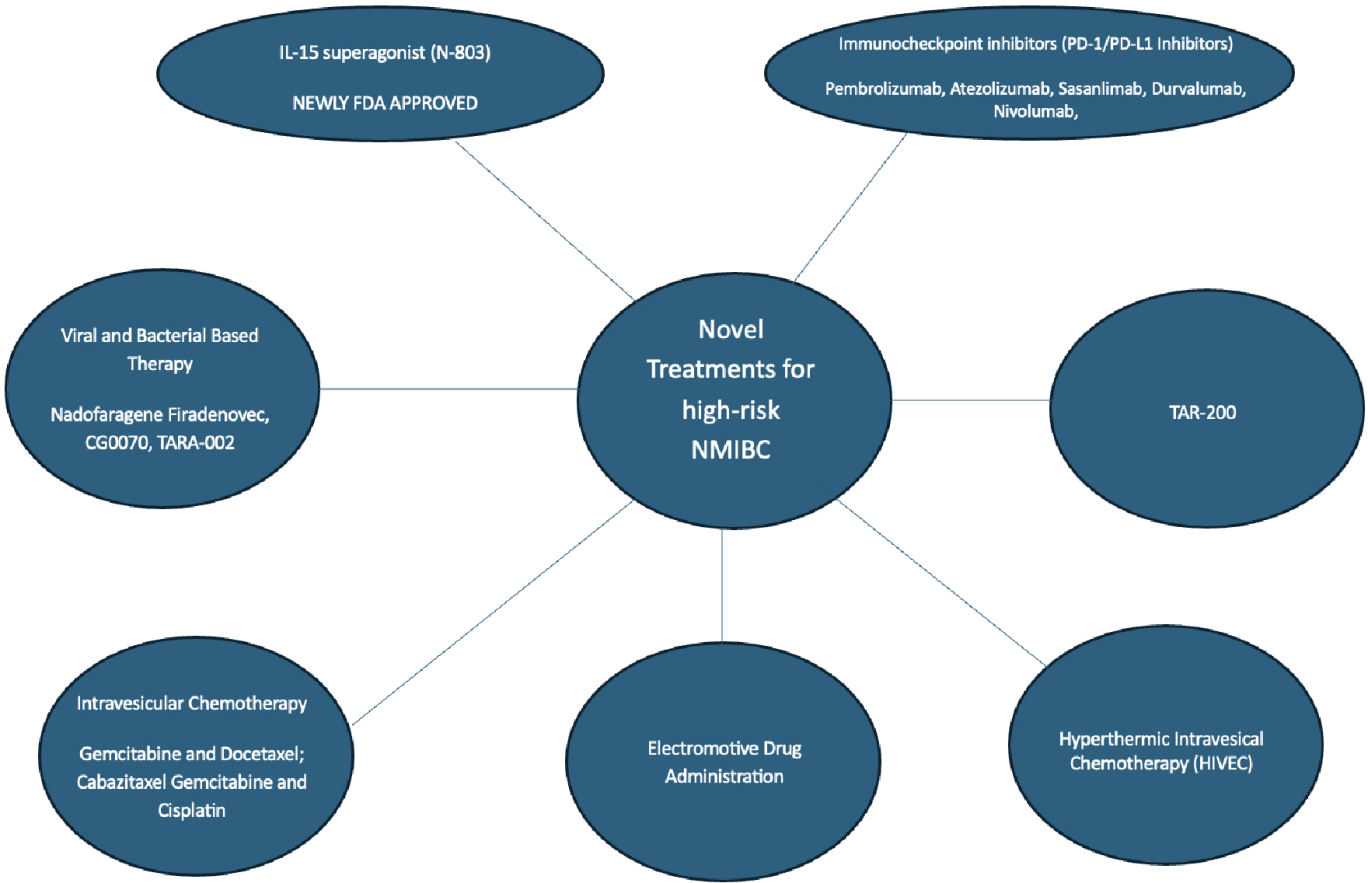
Novel treatments for high-risk non-muscle invasive bladder cancer.

Bacillus Calmette-Guerin (BCG) has been the cornerstone of intravesical therapy given its ability to decrease progression and recurrence of NMIBC after surgical resection or ablation (10). There are scenarios where treatment with BCG is unsuccessful and is no longer a treatment option for the management of high-risk NMIBC including BCG failure (where muscle invasive bladder cancer is detected), BCG-refractory (detection of high-risk lesions during or after adequate treatment at 3 or 6 months of treatment), and BCG-relapsing (detection of tumor following initial response after completion of treatment) (4). Considering the recent global BCG shortage, there is an unmet need for novel treatments of high-risk NMIBC (11). As a result, there have been many new studies and treatments to address the management of BCG unresponsive NMIBC. The current gold standard for treatment following unsuccessful BCG therapy is a radical cystectomy. For patients unwilling or unfit to undergo cystectomy, intravesical Nadofaragene Firadenovec and systemic Pembrolizumab were previously the only two FDA options for BCG-unresponsive NMIBC. As of April 23, 2034, the FDA has approved the immunotherapy boosting drug, N-803, which is marketed under the brand name Anktiva, to be used in combination with the immunotherapy Bacillus Calmette-Guerin (BCG) for the treatment of patients with BCG-unresponsive non–muscle-invasive bladder cancer. This review encompasses studies that are either published in peer-reviewed journals or presented at national meetings and presents the updated novel treatments and ongoing trials for high-risk NMIBC. We categorize treatments according to their main mechanism of action (**Figure 1**).

## IL-15 superagonist

### N-803 (Anktiva), FDA approved

The FDA has recently approved Anktiva (also known as N-803 or Nogapendekin alfa inbakicept) for the treatment of bladder cancer. N-803 was developed to improve the immune-mediated effects of interleukin-15, allowing for the activation and proliferation of natural killer and CD8 + T cells, and avoiding regulatory T cell stimulation. This can be upregulated and ultimately boost the immune response caused by BCG for the treatment of NMIBC (12). Anktiva is specifically approved for patients with Bacillus Calmette-Guerin (BCG)-unresponsive NMIBC. N-803 administration follows standard BCG administration and is done simultaneously. Clinical trials (Quilt 3.302) demonstrated that Anktiva, in combination with BCG, had increases in the complete response rate and durability of response in patients. There was no comparison arm with BCG monotherapy as FDA approval was granted without it. The authors recently reported on two cohorts: (1) those with CIS with or without papillary tumors (n = 83) and (2) patients with papillary tumors only (n = 77). The complete response rate in the CIS cohort was 71% with a median duration of 24 months, whereas in the papillary group, the disease-free rate was 57% and 48% at 1 and 2 years, respectively. In total, 91% and 95% of patients in the CIS and non-CIS cohorts avoided cystectomy, respectively. No treatment-related grades 4 or 5 were reported (13).

A recent open-label, multicenter study evaluated the N-803 in patients with Bacillus Calmette-Guérin (BCG)-unresponsive non-muscle-invasive bladder cancer (NMIBC). Participants with carcinoma *in situ* (CIS), with or without Ta/T1 papillary disease, received intravesical N-803 combined with BCG (cohort A) or N-803 alone (cohort C), while those with high-grade Ta/T1 papillary NMIBC received N-803 plus BCG (cohort B). The results showed a complete response (CR) rate of 71% at 3 months in cohort A and a durable CR rate of 58% at 12 months. For cohort B, the 12-month disease-free survival (DFS) rate was 57%, highlighting significant clinical benefits. Overall, the combination of NAI and BCG demonstrated superior efficacy in high-risk BCG-unresponsive NMIBC, providing a promising therapeutic option with manageable safety profiles (13). These findings emphasize the potential of NAI to improve outcomes for this challenging patient population (13).

## Immune checkpoint inhibitors (PD-1/PD-L1 Inhibitors)

### Pembrolizumab, FDA approved

Pembrolizumab (Keytruda) has emerged as a significant treatment option for patients with NMIBC that is unresponsive to Bacillus Calmette-Guérin (BCG) therapy, particularly those with high-risk carcinoma *in situ* (CIS). Approved by the FDA in January 2020, pembrolizumab is indicated for patients who are either ineligible for or have opted not to undergo cystectomy (14, 15). Pembrolizumab is given either every 3 weeks or 6 weeks. Therapy is continued until disease progression, unacceptable toxicity, or up to 24 months in patients without disease progression (16).

The efficacy of pembrolizumab in this setting was primarily demonstrated in the KEYNOTE-057 trial. In this study, 96 patients with BCG-unresponsive CIS received pembrolizumab at a dose of 200 mg every three weeks. The results showed a complete response rate of 41%, with a median response duration of 16.2 months. Notably, 46% of the responders maintained their response for at least one year (16).

Combination treatment of pembrolizumab with BCG is currently being assessed by KEYNOTE-676, where patients with persistent or recurrent high-risk disease after only one BCG induction course are enrolled to either receive pembrolizumab plus BCG or additional BCG monotherapy. The KEYNOTE-676 is expected to finish by the end of 2024 (17).

Overall, pembrolizumab represents a viable therapeutic alternative for managing high-risk NMIBC, providing a meaningful response rate and duration of response for patients who have limited options following BCG therapy.

### Atezolizumab

Atezolizumab, a PD-L1 inhibitor, is being investigated for its role in treating NMIBC particularly in patients who are unresponsive to Bacillus Calmette-Guérin (BCG) therapy. The SWOG S1605 trial, a single-arm phase II study, evaluated the efficacy of atezolizumab in patients with high-risk BCG-unresponsive NMIBC. Patients received atezolizumab intravenously every three weeks for one year. The trial included 166 patients in the safety analysis and 129 in the efficacy analysis. The results showed that 27% of patients with carcinoma *in situ* (CIS) achieved a complete response at six months. 9.3% of patients experienced progression to muscle invasive or metastatic disease. Overall, the efficacy was considered modest with limited event-free survival rates and needs to be carefully balanced with significant risk of side effects and disease progression (18).

Another trial, BladderGATE, a phase Ib/II study, combined atezolizumab with intravesical BCG in BCG-naïve high-risk NMIBC patients. This combination aimed to leverage the immune-boosting properties of both treatments. Preliminary results indicated that this combination was safe and showed potential efficacy. There was only 14% of 2-years local recurrence-rate and 8% of local progressive-disease which are promising results, pending to randomized Ph3 ALBAN study data (GETUG). Ultimately this suggests that atezolizumab may enhance the therapeutic effects of BCG in NMIBC​ (19).

### Sasanlimab

Sasanlimab, an anti-PD-1 monoclonal antibody, is being studied for its potential in treating NMIBC particularly in patients who are unresponsive to Bacillus Calmette-Guérin (BCG) therapy. The CREST study is a significant trial exploring the efficacy and safety of sasanlimab for high-risk NMIBC. This phase III trial includes multiple cohorts, with one cohort focusing on sasanlimab as monotherapy for BCG-unresponsive patients and another combining sasanlimab with BCG in BCG-naïve patients​ (20). Sasanlimab is typically administered subcutaneously once every 2 weeks or as specified in clinical trial protocols. The duration of therapy is dependent on the response.

Preliminary results from phase Ib/II studies have shown that subcutaneous administration of sasanlimab is safe and has a favorable benefit-risk profile, regardless of baseline tumor PD-L1 levels. The CREST trial aims to determine whether sasanlimab can improve outcomes such as event-free survival and complete response rates compared to BCG alone​.

Overall, sasanlimab represents a promising new option for patients with high-risk NMIBC, especially those who have limited options following BCG therapy failure. Ongoing studies will provide more definitive evidence regarding its role and efficacy in this setting.

### Durvalumab

Durvalumab, a monoclonal antibody targeting PD-L1, is being investigated for its role in treating NMIBC, particularly in patients with high-grade NMIBC that is unresponsive to Bacillus Calmette-Guerin (BCG) therapy. Several studies have explored its safety, tolerability, and efficacy in combination with other agents. Durvalumab is typically administered every two weeks or as specified in clinical trials protocols. Treatment continues until one of the following: disease progression, unacceptable toxicity, or completion of a maximum treatment duration, typically 12-24 months.

One phase I trial evaluated the combination of durvalumab and Vicineum in subjects with high-grade NMIBC previously treated with BCG. The primary objectives were to assess safety and tolerability, while secondary objectives included evaluating efficacy, pharmacokinetics, and biomarker analysis. Initial results showed that the combination was generally well-tolerated, with no dose-limiting toxicities observed in the initial safety cohort. Most patients experienced treatment-related adverse events (TRAEs), with some experiencing grade 3 or higher TRAEs such as hematuria and bladder infections (21).

Another study examined durvalumab combined with BCG in BCG-naive patients with high-risk NMIBC (PATAPSCO Trial). This study aimed to assess the safety and potential efficacy of this combination therapy. Preliminary findings indicated that the combination was feasible, with manageable safety profiles and promising activity in preventing disease recurrence and progression.

There are also many early studies testing the feasibility of intravesical durvalumab. The Hellenic GU Cancer group conducted a phase II clinical trial investigating the feasibility of intravesical administration of durvalumab in patients with high-risk non-muscle-invasive bladder cancer (NMIBC) following Bacillus Calmette-Guérin (BCG) failure. The study aimed to assess the safety and efficacy of this treatment approach. The results indicated that intravesical durvalumab is feasible and exhibits an excellent safety profile, with no grade 3 or higher toxicities reported. Efficacy outcomes were promising, with a one-year high-grade relapse-free rate of 38.5%, a six-month high-grade recurrence-free rate of 50%, and a one-month high-grade recurrence-free rate of 100%. These findings suggest that intravesical immunotherapy using durvalumab could be a viable bladder-preserving strategy for patients with high-risk NMIBC after BCG failure, warranting further investigation in larger clinical trials (22).

Overall, durvalumab shows promise as part of combination therapies for treating high-risk NMIBC, particularly in patients who have not responded adequately to BCG. Further studies and ongoing clinical trials will provide more comprehensive data on its long-term efficacy and safety.

### Nivolumab

Nivolumab, a PD-1 immune checkpoint inhibitor, has shown promise in treating NMIBC, particularly for patients unresponsive to Bacillus Calmette-Guérin (BCG) therapy. Clinical trials, such as CheckMate 9UT, are evaluating its efficacy and safety in combination with BCG or linrodostat, an IDO1 inhibitor. Early results suggest that nivolumab can induce meaningful clinical responses and manageable safety profiles in patients with high-risk, BCG-unresponsive NMIBC (23). Nivolumab is typically administered intravenously every 2-4 weeks or as specified in clinical trials protocols. Duration of therapy is dependent on response.

In the CheckMate 274 study, nivolumab was used as an adjuvant treatment post-surgery for high-risk muscle-invasive bladder cancer, showing significant improvements in disease-free survival, especially in patients whose tumors expressed PD-L1​. However, its application in NMIBC is still under investigation, with ongoing trials assessing its potential to prevent disease progression and recurrence​.

For NMIBC specifically, nivolumab is being tested in combination with BCG to explore whether this combination can enhance the immune response against cancer cells that have persisted or recurred after BCG treatment alone​ (24). The outcomes of these studies could offer new therapeutic avenues for patients with limited options, emphasizing the role of immunotherapy in early-stage bladder cancer management.

## Viral and bacterial based therapy

### Nadofaragene firadenovec (FDA approved)

Nadofaragene firadenovec, marketed as ADSTILADRIN, is an FDA-approved intravesical gene therapy for treating Bacillus Calmette-Guérin (BCG)-unresponsive NMIBC. This therapy is specifically indicated for patients with high-risk NMIBC with carcinoma *in situ* (CIS) with or without papillary tumors who have not responded to BCG treatment. Nadofaragene firadenovec consists of two parts, a recombinant adenovirus vector-based gene therapy delivering a copy of the human interferon alfa-2b gene to urothelial cells, and polyamide surfactant that enhances the viral transduction of the urothelium (25).

In a phase 3 single-arm multicenter study in the USA, Boorjan et al. include 157 patients with BCG-unresponsive NMIBC, who received at least one dose of intravesical nadofaragene firadenovec. This study demonstrating promising results, with a complete response (CR) rate of 53% in patients with BCG-unresponsive CIS. The median duration of response was approximately 9.7 months, with a significant portion of patients remaining in CR for over a year. Long-term data indicate sustained efficacy, with about 34.2% of patients maintaining a durable response for at least three years. Safety profiles from these studies suggest that the treatment is well tolerated, with most adverse events being mild to moderate in severity​ (26).

This gene therapy represents a significant advancement for patients with NMIBC, particularly for those who have limited options beyond cystectomy. It provides a non-surgical alternative that can effectively manage high-risk bladder cancer, offering new hope for long-term disease control.

### Cretostimogene Grenadenorepvec

Cretostimogene Grenadenorepvec (CG0070) is a modified adenovirus that expresses granulocyte–macrophage colony-stimulating factor (GM-CSF) in malignant bladder cells that have a deficient or mutated expression of the retinoblastoma tumor suppressor gene. As such, CG0070 induces cell lysis and augments the immunogenic response with the GM-CSF (27).

In the phase 2 CORE1 study, CG0070 was combined with pembrolizumab, an anti-PD-1 therapy, in patients with BCG-unresponsive NMIBC. The study enrolled 35 patients, and the combination therapy demonstrated a high complete response (CR) rate. At the initial three-month assessment, 88% of evaluable patients achieved CR, with a sustained response rate of 73% at 12 months (28).

The BOND-003 trial is a phase 3, single-arm study evaluating the efficacy and safety of intravesical CG070 in patients with high-risk, BCG- unresponsive NMIBC. The study enrolled 105 patients, with updated results indicating a 75.2% complete response rate at any time based on central review. Notably, 53.8% of patients who underwent repeat induction achieved a complete response. Durability of response was demonstrated, with 52 patients maintaining a response for at least 6 months, 29 patients for 12 months, and 14 patients for 21 months. The 12-month progression-free survival rate was 96.7%, and the cystectomy-free survival rate was 92.4%, with no patients undergoing radical cystectomy or experiencing nodal or metastatic progression among those who achieved a complete response. CG070 was generally well-tolerated, with most adverse events being grade 1-2; only 1.8% of patients experienced serious treatment-related adverse events, and no grade ≥3 treatment-related adverse events were reported. Based on the results of BOND-003, the FDA has granted fast track designation for cretostimogene monotherapy in BCG-unresponsive CIS with or without Ta/T1 papillary disease (29).

The PIVOT-006 study is a phase 3, randomized trial evaluating the efficacy and safety of CG0070 in combination with pembrolizumab for patients with intermediate-risk non-muscle-invasive bladder cancer. As of January 2025, the PIVOT-006 study is still ongoing, and detailed results have not yet been published (30).

These results indicate that CG0070, particularly when combined with immune checkpoint inhibitors like pembrolizumab, holds significant potential as a bladder-sparing treatment option for patients with high-risk, BCG-unresponsive NMIBC. Further studies and longer follow-up will help to solidify its role in clinical practice. Further studies and longer follow-up will help to solidify its role in clinical practice.

### TARA-002

TARA-002 is a lyophilized mixture of low-virulence Streptococcus pyogenes cells that are treated with benzylpenicillin. TARA-002 is a broad immunopotentiator, that is hypothesized to activate both the innate and adaptive immune systems with tumor cells.

In the ADVANCED-1 trial, TARA-002 demonstrated anti-tumor activity and favorable safety profiles across multiple dose levels, with most adverse events being mild to moderate in severity. Among nine patients in this trial, those with carcinoma *in situ* (CIS) showed tumor regression, and one heavily pre-treated, BCG-unresponsive patient achieved a complete response (31).

In the ADVANCED-2 trial, up to 102 patients with BCG-naïve and BCG-unresponsive NMIBC with CIS are being treated with TARA-002. Preliminary results from this trial are expected later in 2024, aiming to further validate the safety and efficacy observed in earlier studies. The trial includes both induction and maintenance therapy phases to evaluate long-term outcomes​.

These studies suggest that TARA-002 may be a valuable addition to the treatment options for NMIBC, particularly for patients who are unresponsive or unable to receive BCG therapy. Further research and upcoming trial results will provide more insights into its potential role in managing this challenging condition.

### Enfortumab vedotin

Enfortumab vedotin is an antibody-drug conjugate targeting Nectin-4, a protein commonly expressed in urothelial carcinoma. Kamat et al. have performed a phase 1 clinical trial assessing the safety and efficacy of intravesical administration of enfortumab vedotin in NMIBC patients. The study enrolled patients with high-risk NMIBC who were unresponsive to Bacillus Calmette-Guérin (BCG) therapy. The primary objectives were to evaluate the safety profile, determine the maximum tolerated dose, and assess preliminary anti-tumor activity. The results demonstrated that intravesical EV was well-tolerated, with manageable adverse events, and showed promising anti-tumor activity, warranting further investigation in larger clinical trials (32).

### Belzupacap sarotalocan

Belzupacap sarotalocan is a novel agent that combines tumor-targeting with photoactivation, selectively binding to cancer cells expressing glypican-3 (GPC3) and inducing cell death upon exposure to light from laser use. AU-001 was an 1, open-label trial to evaluate feasibility and safety of intramural injection of belzupacap sarotalocan. Results from the phase 1 portion demonstrated that the therapy was well-tolerated, with manageable adverse events. Notably, complete responses were observed in a subset of patients, with promising anti-tumor activity. In the phase 2 portion, patients showed durable responses, and the therapy effectively targeted tumor cells while minimizing systemic exposure, supporting further investigation of belzupacap sarotalocan as a potential bladder-preserving treatment option for high-risk NMIBC patients (33).

## Intravesical chemotherapy

### Gemcitabine and docetaxel

Gemcitabine and docetaxel have shown promise as a treatment option for NMIBC, particularly in high-risk patients and those unresponsive to Bacillus Calmette-Guérin (BCG) therapy. The combination involves sequential intravesical administration, where gemcitabine is first instilled into the bladder, followed by docetaxel.

Steinberg et. Al. have demonstrated encouraging results for this regimen. Their retrospective 2020 study involving 276 patients with recurrent NMIBC revealed that the gemcitabine-docetaxel combination provided a recurrence-free survival (RFS) of 60% at one year and 46% at two years, with high-grade recurrence-free survival rates of 65% at one year and 52% at two years. The study highlighted that this combination could be effective even in patients with carcinoma *in situ* and those classified as BCG-unresponsive​ (34).

McElree et. Al. retrospectively compared the efficacy of gemcitabine-docetaxel to BCG in 312 high-risk, treatment-naïve NMIBC patients. This study found improved outcomes with the combination therapy. The high-grade RFS at six months was 92% for gemcitabine-docetaxel versus 76% for BCG, and at two years, it was 81% versus 69%, respectively. Progression-free survival, cystectomy-free survival, and cancer-specific survival rates were also higher in the gemcitabine-docetaxel group (35).

These findings suggest that gemcitabine and docetaxel could serve as a feasible alternative to BCG, especially during BCG shortages, and support ongoing trials like the BRIDGE study, which aims to provide further comparative data.

### Cabazitaxel, gemcitabine, and cisplatin

In a Phase 1 trial, DeCastro Et. Al. studied patients with BCG-unresponsive or recurrent NMIBC who either declined or were ineligible for radical cystectomy. These patients were treated with a 6-week induction regimen of sequentially administered cabazitaxel, gemcitabine, and cisplatin. This study aimed to assess the safety and determine the maximum tolerated dose for these drugs when used intravesical. Preliminary results suggested that this regimen is feasible and demonstrated manageable safety profiles, laying the groundwork for further efficacy studies (36).

Following these encouraging results, a Phase 2 trial is currently underway to evaluate the efficacy of the CGC combination. This trial aims to determine the overall response rate, particularly focusing on the complete response rate, which is defined as no detectable cancer on post-treatment evaluations. The study hopes to provide a viable salvage therapy option for high-risk NMIBC patients who do not respond to BCG and is currently enrolling subjects (37).

This combination therapy leverages the synergistic effects of these chemotherapeutic agents to potentially enhance treatment efficacy and improve outcomes for NMIBC patients.

## Electromotive drug administration

Electromotive Drug Administration (EMDA) is a technique used in the treatment of NMIBC. It involves the delivery of chemotherapy drugs directly into the bladder using a mild electric current to enhance drug penetration. This method aims to increase the efficacy of the chemotherapy while minimizing systemic side effects.

Based on a prior randomized control trial suggesting improved efficacy of sequential EMDA of mitomycin C and BCG in the treatment of BCG-naïve T1 disease (38), Juvet ET. Al. recently assessed the role of this modality in 22 high-risk BCG-unresponsive patients and four others who had received an induction course of BCG (39). The primary study outcome was progression-free survival. The investigators reported a progression-free survival of 58.3% and 48.9% at 1 and 2 years, respectively, whereas the recurrence-free rates were 41.9% and 27.2% at 1 and 2 years, respectively. The complete response rate was 44% at 1 year and 30.4% at 18 months.

Research indicates that EMDA can improve the response rate and reduce the recurrence of NMIBC compared to traditional methods of drug instillation. Studies have shown that EMDA with mitomycin C, a commonly used chemotherapy agent, leads to better outcomes in terms of tumor recurrence and progression. Of note, EMDA is currently not available in the United States.

## Hyperthermic intravesical chemotherapy

Hyperthermia is thought to optimize pharmacokinetics of intravesical chemotherapy by increasing the permeability of the cellular membrane of the bladder leading to better drug absorption into the bladder wall, hyperactivation of the immune system and inhibition of angiogenesis (40). Pijpers et al. performed a retrospective review of patients receiving HIVEC which was limited to intravesical mitomycin C heated to 42° C. The study reported a high-grade recurrence-free rate of 53% and 35% at 1 and 2 years, respectively following treatment. The complete response rate in patients with CIS was 70% at 6 months, and only one patient in their study developed a serious adverse event (grade 3 — urinary tract infection). Tan Et.Al. performed and randomized control trial for patients treated with HIVEC or room temperature Mitomycin C (41). The study reported 32% of patients in the control group had recurrence and 38% of patients in the HIVEC group had recurrence. Disease-free survival at 24 months was 61 in the HIVEC arm and 60% in the control arm. Overall survival was similar between the two groups and patients undergoing HIVEC were less likely to complete their therapy. The study concluded that HIVEC cannot be recommended over chemotherapy alone for intermediate-risk NMIBC. Adverse events following HIVEC were of low grade and short-lived, although patients were less likely to complete their treatment. The adoption of this treatment modality has been limited, as more recent studies have stopped accrual for various reasons and currently there are no currently active trials in the USA.

## TAR-200

TAR-200 is an investigational drug delivery system that releases a controlled, continuous dose of gemcitabine. TAR-200 utilizes an implanted indwelling intravesical device. Ultimately, TAR-200 increases the amount of time the drug delivery system spends in the bladder and sustains local drug exposure. The safety and efficacy of TAR-200 are being evaluated in Phase 2 and Phase 3 studies in patients with muscle-invasive bladder cancer in SunRISe-2 and SunRISe-4 and NMIBC in SunRISe-1 and SunRISe-3. Currently, TAR-200 and cetrelimab (PD-1 inhibitor) are being evaluated in a randomized multicenter phase 2b study in patients in BCG-unresponsive NMBIC. The primary outcome of the study is complete response following treatment with either TAR-200 with cetrelimab (Cohort 1, C1), TAR-200 alone (Cohort 2, C2), or cetrelimab alone(Cohort 3, C3) (42). Preliminary results of the SunRISe-1 study included 23 evaluable patients in C2 and 24 evaluable patients in C3. After median follow-up of 10.6 months, 15 of 16 responses in C2 are still ongoing; median duration of response (DOR) was not reached. Additionally, six of the patients in C2 maintained their response beyond 12 months and none of the complete responders had documented recurrence or progression. The initial findings from SunRISe-1 showed low rates of grade three or higher adverse events (AEs) and a limited number of treatment discontinuations due to adverse events were observed with TAR-200 (43). SunRISe-3 is currently recruiting patients for their studying analyzing TAR-200 in combination with Cetrelimab or TAR-200 alone versus intravesical BCG in patients with BCG-naïve High risk NMIBC with an estimated completion date in 2029.

## Conclusions

The BCG shortage has emphasized the urgent need for alternative treatment options for high-risk NMIBC where BCG has been a cornerstone therapy. While radical cystectomy remains the gold standard for BCG-unresponsive NMIBC, it is a highly invasive procedure with significant morbidity (6). Efforts by urologic societies have led to the FDA’s acceptance of single-arm trials, accelerating drug development for this challenging disease. In the past few years, the FDA has approved multiple new options including pembrolizumab, Nadofaragene Firadenovec, intravesical valrubicin, and most recently, N-803. In this review we have detailed several novel treatment options with potential for FDA approval (**Table 2**).

**Table 2:** Summary of novel NMIBC therapies, clinical trials, and associated results of the trials.

Drug	FDA Approval	Study	Mechanism of Action	Number of Pts in Study	Response Rate	Reported Response Duration	Cystectomy free rate
Anktiva (N-803)	Yes	Quilt 3.302	Activation and proliferation of natural killer and CD8 + T cells	CIS (n=83); nonCIS (n=77)	CIS 71%; nonCIS 48%	24 months	CIS 91%; nonCIS 95%
Pembrolizumab	Yes	KEYNOTE-057	Monoclonal antibody binds to PD-1 inhibiting interaction with PD-L1 and PD-L2	96	41%	16.2 months	Not Reported
Atezolizumab	No	SWOG S1605	PD-L1 inhibition	129	27%	6 months	Not Reported
Sasanlimab	No	CREST	Monoclonal antibody binds to PD-1 inhibiting interaction with PD-L1 and PD-L2	Preliminary results not reported	Not yet reported	Not yet reported	Not yet reported
Durvalumab	No	PATAPSCO	Monoclonal antibody binds PD-L1 inhibiting its interaction with PD-1	Preliminary results not reported	Not yet reported	Not yet reported	Not yet reported
Nivolumab	No	CheckMate 9UT	PD-1 immune checkpoint inhibitor	Preliminary results not reported	Not yet reported	Not yet reported	Not yet reported
Nadofaragene Firadenovec	Yes	INSTILADRIN	Recombinant adenovirus vector and polyamide surfactant	157	a) 53% b) 34.2%	a) 9.7 months b) 36 months	Not Reported
CG0070	No	CORE1	Modified adenovirus that induces cell lysis paired with anti PD-1 therapy	35	a) 88% b) 73%	a) 3 months b) 12 months	Not Reported
TARA-002	No	ADVANCED-1	Lyophilized mixture of low-virulence Streptococcus pyogenes cells treated with benzylpenicillin	102	Not reported	Not yet Reported	Not yet reported
Gemcitabine & Docetaxel	No	Retrospective Review	Combination intravesical therapy	276	a) 60% b) 46%	a) 12 months b) 24 months	Not Reported
Cabazitaxel, Gemcitabine, Cisplatin	No	Phase 2 trial	Combination intravesical therapy	Preliminary results not reported	Not yet reported	Not yet reported	Not yet reported
Electromotive Drug Administration	No	Prospective Trial	Intravesical chemotherapy with mild electric current to enhance penetration	26	a) 44% b) 30.4%	a) 12 months b) 18 months	Not Reported
Hyperthermic Intravesical Chemotherapy	No	Retrospective Review	Heated intravesical therapy	56	a) 53% b) 35%	a) 12 months b) 24 months	Not Reported
TAR-200	No	SUNRISE-1 and SUNRISE-2	Investigational drug delivery system; controlled, continuous dose of gemcitabine or cetrelimab	23	Not yet reported	Not yet reported	Not yet reported

As the horizon of NMIBC treatment continues to change, it is crucial to consider multiple patients factors before proceeding with treatment including medication cost, goals of care, delay in potential surgery, and adverse medication effects. For example, the economic burden of pembrolizumab can amount to roughly $12,500 monthly while the Keynote trial reported approximately 20% complete response at 12 months. Clinicians most be able to strike this balance with patients while discussing treatment options. Moreover, the lack of randomized trials poses challenges for direct comparisons of these new treatments, which is critical for clinical decision-making.

In our clinical practice, BCG-unresponsive disease is encountered frequently, particularly in patients who are either unfit or unwilling to undergo radical cystectomy. Thus, we strongly advocate for participation in clinical trials. We believe that clinical trial enrollment is essential for advancing the treatment landscape of NMIBC and improving patient outcomes. The continued development and rigorous evaluation of these innovative therapies hold promise for enhancing the quality of care for bladder cancer patients.
